# Supplementary material for: The Evolution and Characterization of the RNA Interference Pathways in Lophotrochozoa
Source: Genome Biol Evol. 2024 May 7;16(5):evae098. doi: 10.1093/gbe/evae098 (PMC11114477; doi:10.1093/gbe/evae098)
Supplement: evae098_Supplementary_Data [file evae098_supplementary_data.zip › Supplementary_materials.zip!/selected_miRNAs/NoGe_selected_miRNAs/mirdeep_pdf_files/NMRB01000231.1_Notospermus_geniculatus_strain_Ushimado_scaffold231,_whole_genome_shotgun_sequence_7813.pdf]

Provisional ID	:	NMRB01000231.1_Notospermus_geniculatus_strain_Ushimado_scaffold231,_whole_genome_shotgun_sequence_7813
Score total	:	1721
Score for star read(s)	:	3.9
Score for read counts	:	1712.1
Score for mfe	:	1.9
Score for randfold	:	0
Score for cons. seed	:	3
Total read count	:	3370
Mature read count	:	3164
Loop read count	:	0
Star read count	:	206

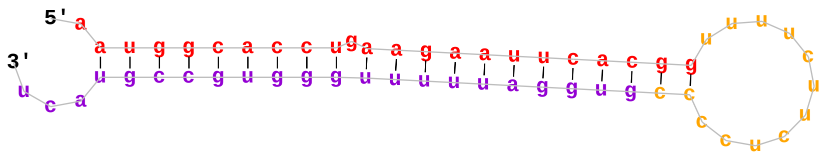

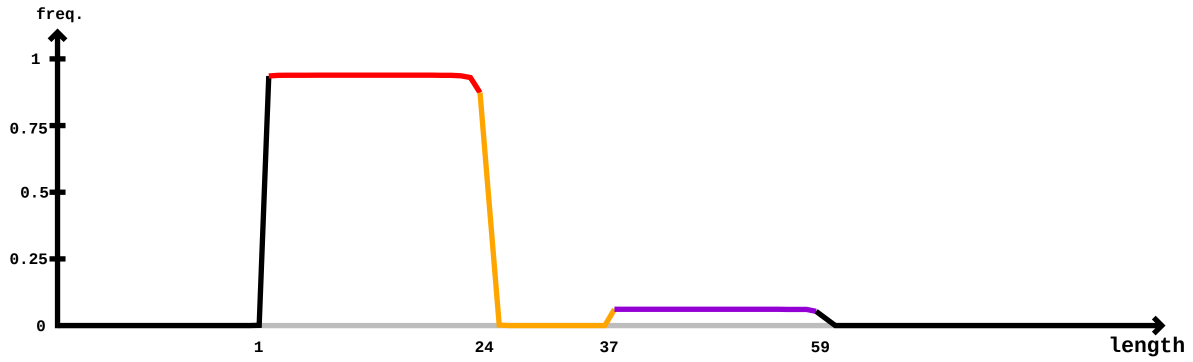


```
                    Mature                            Star
5'- gcuuugcuuuugucuccaagaauggcaccugaagaauucacgguuuucuucuccccguggauuuuugggugccguacuuggguugaugguuuuucaucacugagccauguuuc   -3'    obs
    gcuuugcuuuugucuccaagaauggcaccugaagaauucacgguuuucuucuccccguggauuuuugggugccguacuuggguugaugguuuuucaucacugagccauguuuc          exp
    ..............((((((.(((((((((.((((((((((((...........))))))))))))))))))))).))))))...(((((((.........))))))).....   reads    mm      sample
    ....................aauggcaccugaagaauuca.........................................................................   1        0       Mm3
    ....................aauggcaccugaagaauucac........................................................................   4        0       Mm3
    ....................aauggcaccugaagaauucacg.......................................................................   13       0       Mm3
    ....................aauggcaccugaagaauucacgA......................................................................   4        1       Mm3
    ....................aauggcaccugaagaauucacgU......................................................................   1        1       Mm3
    ....................aauggUaccugaagaauucacgg......................................................................   2        1       Mm3
    ....................aauggcaccugaagaauucacgg......................................................................   174      0       Mm3
    ....................aauggcaccugaagaauCcacgg......................................................................   1        1       Mm3
    ....................aauggcaccugaagaauGcacgg......................................................................   1        1       Mm3
    ....................aauggcaccugaagaauucacggu.....................................................................   1        0       Mm3
    ........................................................guggauuuuugggugccguac....................................   1        0       Mm3
    ........................................................guggauuuuugggugccguacu...................................   4        0       Mm3

    ...................Caauggcaccugaagaauucacgg......................................................................   1        1       Fm3
    ....................aauggcaccugaagaauu...........................................................................   1        0       Fm3
    ....................aauggcaccugaagaauucac........................................................................   3        0       Fm3
    ....................aauggcaccugaagaauucacg.......................................................................   22       0       Fm3
    ....................aauggcaccugaagaaCucacgg......................................................................   1        1       Fm3
    ....................aauggcaccugaagaauucacgg......................................................................   29       0       Fm3
    ....................aauggcaccugUagaauucacggu.....................................................................   1        1       Fm3

    ...................Caauggcaccugaagaauucacgg......................................................................   2        1       Mm2
    ....................aauggcaccugaagaauu...........................................................................   1        0       Mm2
    ....................aauggcaccugaagaauuca.........................................................................   4        0       Mm2
    ....................aauggcaccugaagaauucac........................................................................   13       0       Mm2
    ....................aauggcaccugaagaauucacU.......................................................................   2        1       Mm2
    ....................Nauggcaccugaagaauucacg.......................................................................   1        1       Mm2
    ....................aauggcaccugaagaauucacg.......................................................................   101      0       Mm2
    ....................aauggcaccugaagaauuUacgg......................................................................   1        1       Mm2
    ....................aauggcaccuAaagaauucacgg......................................................................   2        1       Mm2
    ....................aauggcaccugaagaauGcacgg......................................................................   14       1       Mm2
    ....................aauggcaccugaagaauAcacgg......................................................................   9        1       Mm2
    ....................aauAgcaccugaagaauucacgg......................................................................   1        1       Mm2
```

```
                    Mature                                 Star
gcuuugcuuuugucuccaagaauggcaccugaagaauucacgguuuucuucuccccguggauuuuugggugccguacuuggguugaugguuuuucaucacugagccauguuuc
....................aauggcaccugaagaaCucacgg......................................................................   7     1   Mm2
....................aauggcaccugaagaaAucacgg......................................................................   4     1   Mm2
....................aauggcaccCgaagaauucacgg......................................................................   2     1   Mm2
....................aauggcaccugaagaauucCcgg......................................................................   1     1   Mm2
....................aauggcaccugaagaauucacgC......................................................................   5     1   Mm2
....................aauggcaccugaagGauucacgg......................................................................   1     1   Mm2
....................aauggcaAcugaagaauucacgg......................................................................   1     1   Mm2
....................aauggcaccugaagaauucacgU......................................................................   1     1   Mm2
....................aauggcaccugaagaaGucacgg......................................................................   4     1   Mm2
....................aauggcaccuCaagaauucacgg......................................................................   4     1   Mm2
....................aauggcaccugaagaauCcacgg......................................................................   14    1   Mm2
....................aUuggcaccugaagaauucacgg......................................................................   1     1   Mm2
....................aauggcaccugaagaauucacgA......................................................................   9     1   Mm2
....................aauggcaccugaUgaauucacgg......................................................................   1     1   Mm2
....................aauggUaccugaagaauucacgg......................................................................   2     1   Mm2
....................aaAggcaccugaagaauucacgg......................................................................   1     1   Mm2
....................aaugUcaccugaagaauucacgg......................................................................   2     1   Mm2
....................Uauggcaccugaagaauucacgg......................................................................   2     1   Mm2
....................aauggcaccugaagaauucaUgg......................................................................   1     1   Mm2
....................aauCgcaccugaagaauucacgg......................................................................   1     1   Mm2
....................aauggcaccugaagaauucacgg......................................................................   2089  0   Mm2
....................aaCggcaccugaagaauucacgg......................................................................   1     1   Mm2
....................aauggcUccugaagaauucacgg......................................................................   1     1   Mm2
....................aauggcaccugaagaauGcacggu.....................................................................   1     1   Mm2
....................aauggcaccugaagaauucacggG.....................................................................   1     1   Mm2
....................aauggcaccugaagaauAcacggu.....................................................................   2     1   Mm2
....................aauggcaccugaagaauucacggu.....................................................................   13    0   Mm2
....................aauggcaccugaagaauucacggA.....................................................................   12    1   Mm2
....................aauggcaccugaagaauucacgguu....................................................................   5     0   Mm2
....................aauggcaccugaagaauucacggAu....................................................................   1     1   Mm2
.....................auggcaccugaagaauucacg.......................................................................   2     0   Mm2
.....................auggcaccugaagaauucacgg......................................................................   6     0   Mm2
......................uggcaccugaagaauucacgg......................................................................   1     0   Mm2
.........................caccugaagaauucacgg......................................................................   1     0   Mm2
........................................................guggauuuuugggugccg.......................................   2     0   Mm2
........................................................guggauuuuugggugccguac....................................   4     0   Mm2
........................................................guggauuuuugggugccguacu...................................   12    0   Mm2
........................................................guggauuuuugggugccguacG...................................   1     1   Mm2

....................aauggcaccugaagaauuca.........................................................................   1     0   Mm1
....................aauggcaccugaagaauucac........................................................................   2     0   Mm1
....................aauggcaccugaagaauucacg.......................................................................   15    0   Mm1
....................aUuggcaccugaagaauucacg.......................................................................   1     1   Mm1
....................aauggcaccugaagaauucacgA......................................................................   5     1   Mm1
....................aauggcaccugaagaauucacgU......................................................................   1     1   Mm1
....................aauggcaccugaagaauucacgg......................................................................   116   0   Mm1
....................aauggcaccugaagaauAcacgg......................................................................   2     1   Mm1
....................aauggcaccugaagaaGucacgg......................................................................   1     1   Mm1
....................aauggcaccugaagaauucacggu.....................................................................   4     0   Mm1

....................aauggcaccugaagaauucacg.......................................................................   24    0   Fm1
....................aauAgcaccugaagaauucacgg......................................................................   1     1   Fm1
....................aauggcaccugaagaauAcacgg......................................................................   1     1   Fm1
....................aauggcacUugaagaauucacgg......................................................................   1     1   Fm1
....................aauggcaccugaagaauucacgg......................................................................   94    0   Fm1
....................aauggcaccugaagaauucacggu.....................................................................   1     0   Fm1
....................aauggcaccugaagaauucacggA.....................................................................   1     1   Fm1
....................aauggcaccugaagaauucacgguu....................................................................   1     0   Fm1
........................................................Auggauuuuugggugccguac....................................   19    1   Fm1
........................................................Auggauuuuugggugccguacu...................................   163   1   Fm1

....................aauggcaccugaagaauucacg.......................................................................   10    0   Fm2
....................aauggcacUugaagaauucacgg......................................................................   1     1   Fm2
....................aauggcaccugaagaauGcacgg......................................................................   3     1   Fm2
....................aauggcaccugaagaauCcacgg......................................................................   2     1   Fm2
....................aauggcaccugaagaaGucacgg......................................................................   1     1   Fm2
....................aauggcaccugaagaauucacgg......................................................................   236   0   Fm2
....................aauggcaccugaagaauucaUgg......................................................................   1     1   Fm2
....................aauggcaccugaagaauAcacgg......................................................................   1     1   Fm2
....................aauggcaccugUagaauucacgg......................................................................   1     1   Fm2
```

```
                    Mature                           Star
gcuuugcuuuugucuccaagaauggcaccugaagaauucacgguuuucuucuccccguggauuuuugggugccguacuuggguugaugguuuuucaucacugagccauguuuc
....................aauggcaccugaagaauucacggu.....................................................................    19    0    Fm2
....................aauggcaccugaagaauAcacggu.....................................................................    1     1    Fm2
....................aauggcaccugaagaauucacggA.....................................................................    5     1    Fm2
```
